# Ultraviolet photochemistry of ethane: implications for the atmospheric chemistry of the gas giants†

**DOI:** 10.1039/d0sc01746a

**Published:** 2020-04-29

**Authors:** Yao Chang, Jiayue Yang, Zhichao Chen, Zhiguo Zhang, Yong Yu, Qingming Li, Zhigang He, Weiqing Zhang, Guorong Wu, Rebecca A. Ingle, Matthew Bain, Michael N. R. Ashfold, Kaijun Yuan, Xueming Yang, Christopher S. Hansen

**Affiliations:** State Key Laboratory of Molecular Reaction Dynamics, Dalian Institute of Chemical Physics, Chinese Academy of Sciences 457 Zhongshan Road Dalian 116023 China kjyuan@dicp.ac.cn xmyang@dicp.ac.cn; Key Laboratory of Functional Materials and Devices for Informatics of Anhui Higher Education Institutions, School of Physics and Electronic Engineering, Fuyang Normal University Fuyang Anhui 236041 China; Department of Chemistry, University College London London WC1H 0AJ UK; School of Chemistry, University of Bristol Bristol BS8 1TS UK mike.ashfold@bristol.ac.uk; Department of Chemistry, Southern University of Science and Technology Shenzhen 518055 China; School of Chemistry, University of New South Wales Sydney NSW 2052 Australia christopher.hansen@unsw.edu.au

## Abstract

Chemical processing in the stratospheres of the gas giants is driven by incident vacuum ultraviolet (VUV) light. Ethane is an important constituent in the atmospheres of the gas giants in our solar system. The present work describes translational spectroscopy studies of the VUV photochemistry of ethane using tuneable radiation in the wavelength range 112 ≤ *λ* ≤ 126 nm from a free electron laser and event-triggered, fast-framing, multi-mass imaging detection methods. Contributions from at least five primary photofragmentation pathways yielding CH_2_, CH_3_ and/or H atom products are demonstrated and interpreted in terms of unimolecular decay following rapid non-adiabatic coupling to the ground state potential energy surface. These data serve to highlight parallels with methane photochemistry and limitations in contemporary models of the photoinduced stratospheric chemistry of the gas giants. The work identifies additional photochemical reactions that require incorporation into next generation extraterrestrial atmospheric chemistry models which should help rationalise hitherto unexplained aspects of the atmospheric ethane/acetylene ratios revealed by the Cassini–Huygens fly-by of Jupiter.

## Abstract 2

The vacuum ultraviolet photodissociation dynamics of ethane provide clues for modelling the atmospheric chemistry of the gas giants.

## Graphical abstract

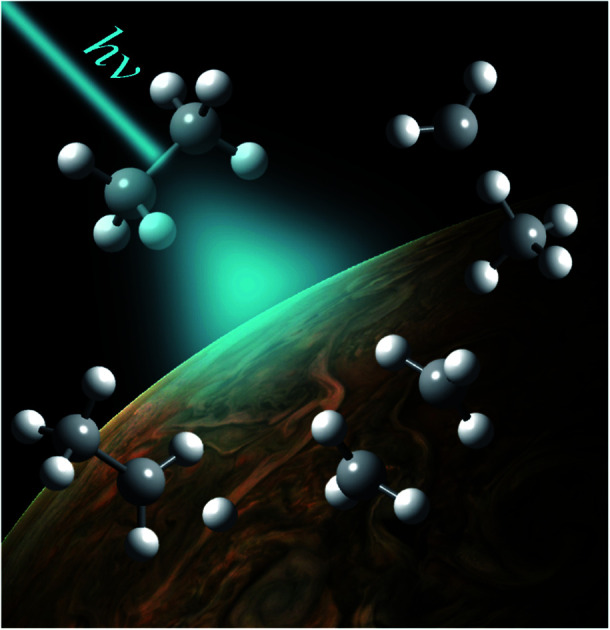

## Introduction

Understanding, and perhaps one day exploiting, the environment of extraterrestrial bodies is a central objective of planetary science. The gas giants in our solar system (Jupiter, Saturn, Uranus and Neptune) are rich in molecular chemistry and remain targets of intense scientific study. Like Earth, each of these planets orbits the sun with its own eccentricity and obliquity leading to seasonal variations in incident solar radiation and thus a cycling chemical composition with latitudinal and altitudinal variations in the abundances of the various molecular constituents.^1^ Absorption of near-infrared solar radiation by methane (CH_4_) makes important contributions to heating the upper atmospheres (stratospheres) of these planets.^1–3^ Methane contributes less to stratospheric cooling, however, which is more reliant on emission from ethane (C_2_H_6_) and acetylene (C_2_H_2_).^1^ Understanding the balance and interplay between CH_4_ and C_2_H_6_/C_2_H_2_ is central to understanding the atmospheric dynamics of the gas giants.

Chemical processing in the stratospheres of the gas giants is driven by incident vacuum ultraviolet (VUV) light,^4^ even in the distant, gas-poor giants Uranus and Neptune.^5^ Numerous possible reactions merit consideration, but common photochemical models for these planetary atmospheres necessarily employ a reduced set pruned from a much larger library of reactions, along with their corresponding rates/branching fractions. These models describe many aspects of the atmospheres of Saturn and Jupiter reasonably well^1–3^ but have recognised shortcomings. For example, the dominant C_2_H_6_ and C_2_H_2_ generation mechanisms are assumed to involve secondary reactions following photolysis of CH_4_.^6–8^ But both the Cassini–Huygens fly-by of Jupiter and terrestrial measurements reveal very different meridional and latitudinal distributions for C_2_H_6_ and C_2_H_2_. Such would be surprising if both species are tightly coupled to methane photolysis.^3,9,10^ Neglect of ion-molecule chemistry has been suggested as one possible explanation for this discrepancy,^3,11,12^ but it is also appropriate to question the inputs to the commonly used photochemical schemes. These draw on data^8^ from a range of (often indirect) sources, including predictions, wherein chemical pathways have been included or removed on the basis of how well the model fits the measurements. Ethane is an important participant in these models and, whilst VUV photolysis is accepted as its main destruction route,^13,14^ the dominant fragmentation pathways and photoproducts are not well determined.

Early laboratory studies of C_2_H_6_ photolysis at the resonance wavelengths emitted by a xenon lamp (*λ* = 147.0 and 129.5 nm) deduced the involvement of (at least) three fragmentation pathways. Two involve loss of H_2_ or two H atoms, the other yields CH_4_ + CH_2_ products.^15^ Subsequent studies using Kr and Ar resonance lamps (*λ* = 123.6 and 106.7/104.8 nm, respectively) suggested additional primary fragmentation channels, to CH_3_ + CH_3_ and, particularly, H + C_2_H_5_ products.^16–18^ These studies all involved careful end-product analysis but could not distinguish primary photofragmentation processes from secondary reactions following photolysis, nor yield any dynamical information. More recent imaging studies showed formation of H atoms following C_2_H_6_ photolysis at the Lyman-α wavelength (*λ* = 121.6 nm, the most intense VUV wavelength in the solar spectrum), with an isotropic velocity distribution peaking at low kinetic energies and a weak tail extending to higher energies. The form of this distribution was attributed to initial C–H bond fission, yielding a fast H atom and an electronically excited 
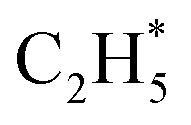
 fragment, followed by a second (slow) H atom from unimolecular decay of the latter.^19^

The present study employs two cutting-edge technologies – the intense, pulsed VUV free electron laser (FEL) at the Dalian Coherent Light Source (DCLS)^20^ and an event-triggered, fast framing, Pixel Imaging Mass Spectrometry (PImMS2) sensor^21^ – to advance understanding of C_2_H_6_ photochemistry and to identify similarities and differences with the photochemistry of both lighter (*i.e*. CH_4_) and heavier (*e.g*. propane (C_3_H_8_)) alkanes. The reported data derive from two sets of collision-free experiments: (i) multi-mass velocity-map ion imaging (PImMS2 detected)^22^ studies following one-color VUV photolysis of ethane and ‘universal’ (*i.e.* not quantum state selected) photoionisation of CH_2_ and CH_3_ photoproducts at four (FEL-produced) wavelengths in the range 112.0 ≤ *λ* ≤ 125.6 nm, and (ii) VUV photolysis at *λ* = 121.6 nm (using photons generated by four wave mixing outputs from a tabletop pulsed laser) and subsequent detection of H atom products using the high resolution H-atom Rydberg tagging technique.^23,24^ The experimental procedures have all been described previously and are thus confined to the ESI.†

## Results and discussion

### (a) Ethane absorption and the energetics of its various dissociation channels

**Fig. 1:**
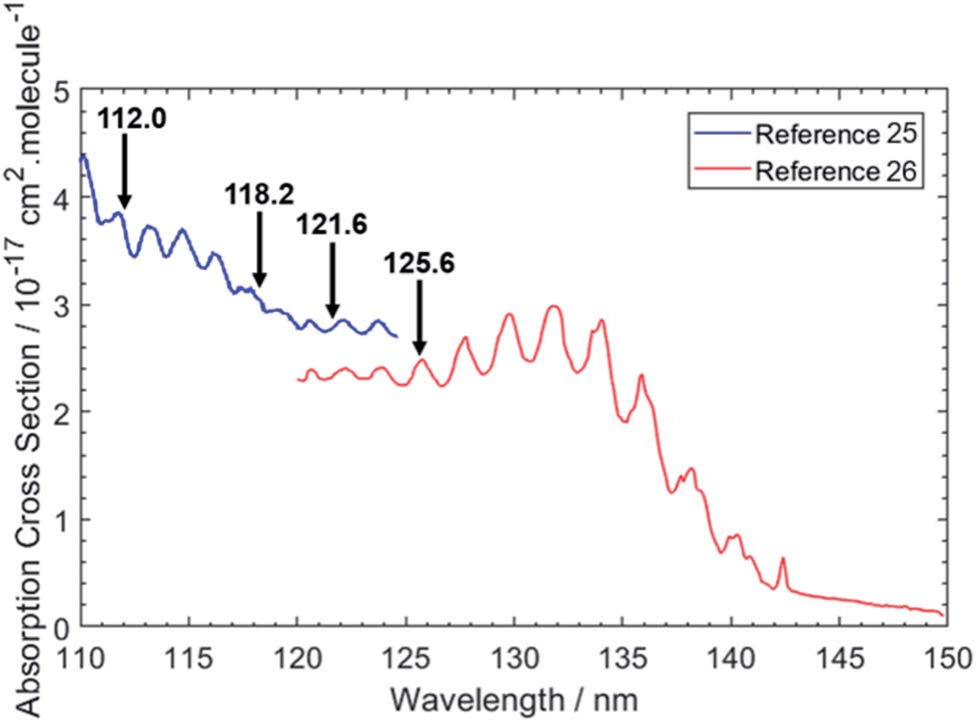
VUV absorption spectrum of C_2_H_6_ from ref. 25 and 26. The data from ref. 26 were extracted manually, while the data from ref. 25 were retrieved from ref. 48. The black arrows indicate the photolysis wavelengths (in nm) used in the present study.

Fig. 1 shows the chosen photolysis wavelengths superimposed on the electronic absorption spectrum of ethane.^25,26^ As with the other alkanes, the absorption of C_2_H_6_ lies in the VUV region but, uniquely amongst the alkanes, its room temperature absorption spectrum displays resolved vibronic structure. This structure is attributed to transitions from the near degenerate highest occupied 3a_1g_ and 1e_g_ valence orbitals to orbitals with dominant 3p Rydberg character. One or more of these are suggested to have significant antibonding valence σ* character also.^27^ Excitations to the 3s Rydberg orbital in C_2_H_6_ are predicted at lower energies, but to be weak – as a result of the molecular center of symmetry – thus distinguishing the 3s Rydberg excitations of ethane from those in CH_4_ and the heavier alkanes which all show large absorption cross-sections. This seemingly simple description hides a wealth of potential complexity, however. The degeneracy of the ground (X̃^2^E_g_) state of the C_2_H_6_^+^ cation is lifted by Jahn–Teller distortion, and the structure and dynamics of the resulting cation states are further complicated by the energetic proximity of the low lying Ã^2^A_1g_ excited state – with the result that even a full interpretation of the threshold photoelectron spectrum of C_2_H_6_ remains elusive.^28^ Such interactions must also affect the Rydberg states of current interest – since they share the same ion core(s) – and thus affect the absorption spectrum shown in Fig. 1.

**Fig. 2:**
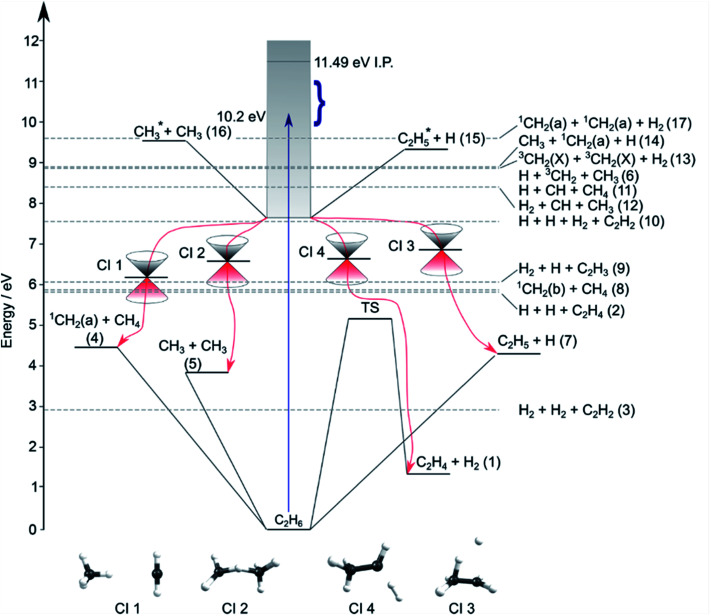
Energy diagram depicting the excited states of ethane, the thresholds for forming various product combinations (as labelled in Table 1) and illustrative members of families of low-lying CIs (labelled CI1–CI4, identified using the GRRM method) that could facilitate non-adiabatic coupling of excited state population to the S_0_ PES and thence to the various dissociation products. Representative structures of these CIs are shown at the foot of the figure. The shaded region indicates the energies spanned by excited electronic states of C_2_H_6_, the density of which increases as the ionisation potential (IP) is approached. The vertical arrow shows the energy of a Lyman-α photon and the bracket indicates the range of photolysis photon energies explored in this study.

Contemporary computational chemistry methods have enabled global investigations of the ground (S_0_) state potential energy surfaces (PESs) for species involved in the early stages of the pyrolysis of ethane and other C_1_–C_3_ hydrocarbons^29^ but have yet to be directed at the excited state photochemistry of any but the very simplest alkanes. Fig. 2 shows the lower-lying dissociation limits of C_2_H_6_. The predicted minimum energy conical intersections were located using the global reaction route mapping (GRRM) method and are discussed later. The S_0_ state correlates adiabatically with the ground state products from either C–C or C–H bond fission (*i.e.* ground state CH_3_ + CH_3_ and H + C_2_H_5_ fragments). The former is the weaker bond, and the formation of ^1^CH_2_ + CH_4_ products is attributed to an (essentially barrierless) H atom transfer between the incipient CH_3_ radicals.^29^ The energetic thresholds for these three processes are all lower than the calculated barrier to H_2_ elimination on the S_0_ PES (∼5.1 eV).^29^ As Fig. 2 also shows, many more spin-allowed fragmentation channels are energetically accessible following electronic excitation of ethane. Table 1 lists no fewer than 17 chemically intuitive channels that require less than the 10.2 eV of energy provided by a Lyman-α photon. Of these, 8, 7, 6 and 5 of the channels yield, respectively, H atoms, H_2_ molecules, CH_2_ and CH_3_ radicals amongst the dissociation products. Such commonalities provide a major challenge for quantitative studies of the primary photochemistry of ethane (and larger alkanes). Of particular relevance to the present study, the reduced models currently used to describe the atmospheric chemistry of Jupiter and Saturn^1–3^ recognise just reactions (1)–(5) in Table 1.

**Table 1:** Possible spin-allowed fragmentation channels for C_2_H_6_ following absorption of a photon with energy *E*_phot_ < 10 eV. Reactions (1)–(5) were used to describe the photoinduced loss of C_2_H_6_ in the recent modelling of the stratospheres of Saturn and Jupiter,^1,3^ reactions (6) and (7) are implicated in the present data interpretation and the remaining reactions are numbered in order of increasing reaction enthalpy (calculated from data in ref. 47)

Products	Δ_r_*H* (0 K)/eV	Dissociation channel
C_2_H_4_ + H_2_	1.34	1
C_2_H_4_ + H + H	5.82	2
C_2_H_2_ + H_2_ + H_2_	3.08	3
CH_4_ + ^1^CH_2_(ã)	4.46	4
CH_3_ + CH_3_	3.81	5
CH_3_ + ^3^CH_2_(X̃) + H	8.55	6
C_2_H_5_ + H	4.30	7
CH_4_ + ^1^CH_2_(b̃)	5.89	8
C_2_H_3_ + H_2_ + H	6.07	9
C_2_H_2_ + H_2_ + H + H	7.56	10
CH_4_ + CH + H	8.40	11
CH_3_ + CH + H_2_	8.40	12
^3^CH_2_(X̃) + ^3^CH_2_(X̃) + H_2_	8.81	13
CH_3_ + ^1^CH_2_(ã) + H	8.94	14
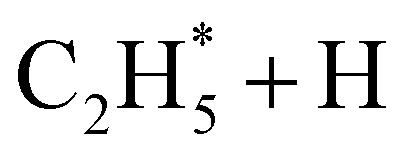	9.33	15
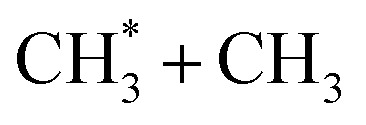	9.54	16
^1^CH_2_(ã) + ^1^CH_2_(ã) + H_2_	9.59	17

### (b) Ion imaging studies

**Fig. 3:**
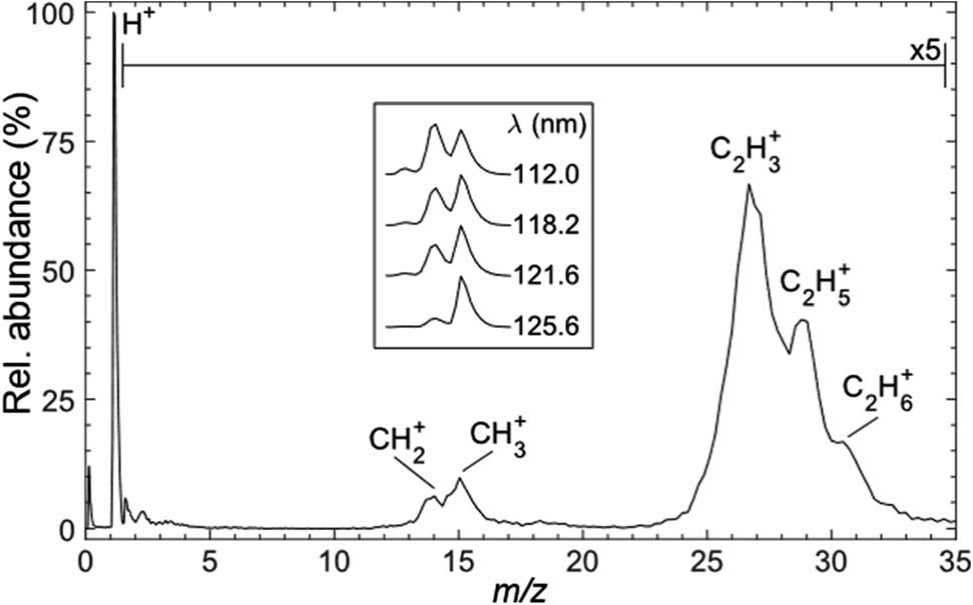
Time-of-flight mass spectrum of ions formed following photoexcitation of a jet-cooled sample of C_2_H_6_ seeded in He at *λ* = 121.6 nm (*hν* = 10.19 eV). Inset: expanded spectra illustrating the *λ*-dependence of the *m*/*z* 14 (CH_2_^+^) and *m*/*z* 15 (CH_3_^+^) peak intensities.

Fig. 3 shows a representative time-of-flight mass spectrum (TOF-MS) of the ions formed following FEL excitation (at *λ* = 121.6 nm) of a jet-cooled sample of C_2_H_6_ in helium. The spectrum is dominated by a peak associated with H^+^ ions. This is unsurprising, given that this wavelength is resonant with the Lyman-α transition from the ground (*n* = 1) state of the H atom. The remainder of the TOF-MS, displayed on a 5× expanded vertical scale, reveals two clumps of partially-resolved peaks corresponding to CH_*x*_^+^ (*x* = 2, 3) and C_2_H_*y*_^+^ (*y* = 3–6) ions. The most intense features in the latter are associated with C_2_H_3_^+^ and C_2_H_5_^+^ ions. Tables S1 and S2 in the ESI† list relevant adiabatic ionisation and dissociative ionisation thresholds, respectively, and show that four of the neutral products of particular interest (*i.e.* CH_2_, CH_3_, C_2_H_3_ and C_2_H_5_) are amenable to single photon ionisation at 10.2 eV, with dissociative ionisation only a (potential) concern if any of these species carry high levels of internal excitation. Note, however, that the observation of some parent C_2_H_6_^+^ ion signal highlights the difficulty of completely excluding multiphoton processes even when operating at threshold FEL pulse intensities (<100 nJ).

The inset to Fig. 3 shows that the relative intensity of the CH_2_^+^ signal increases as the excitation wavelength is decreased. Note that the data shown in the inset were recorded with the detector sensitivity raised for just the relevant narrow range of mass/charge (*m*/*z*) ratios, thus allowing averaging over many more acquisitions and improved signal-to-noise ratios. The *λ*-dependent trend in the CH_2_^+^ signal is also recognisable in spectra recorded using higher FEL pulse energies but, as shown in Fig. S1,† the relative peak intensities are also pulse energy dependent. Such variations are not unexpected, given the inevitable differences in the wavelength and internal energy dependent photoionisation cross-sections for CH_3_, ^1^CH_2_ and ^3^CH_2_ radicals.

Use of the PImMS2 sensor affords not just TOF mass spectra such as those presented in Fig. 3, but also an ion image for each mass channel, in a single acquisition. This provides velocity distributions for each ion peak in Fig. 3. Since C–C bond rupture processes are likely to be pivotal in the cycling of ethane and methane and thus to have a major effect on the atmospheric dynamics, we first present kinetic energy distributions of CH_2_ and CH_3_ fragments (monitored *via* the corresponding ions) from the photofragmentation of ethane.

**Fig. 4:**
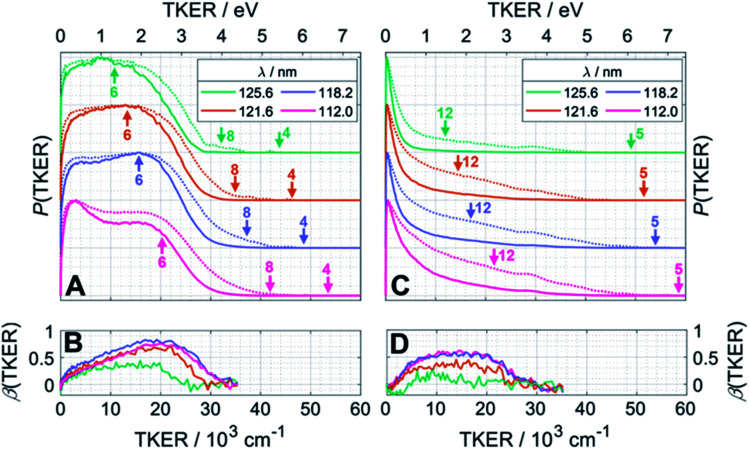
*P*(TKER) and *β*(TKER) distributions derived from the analysis of ion images (Fig. S2†) for (A and B) CH_2_ and (C,D) CH_3_ fragments from photolysis of a jet-cooled C_2_H_6_ sample, with the TKER shown in both cm^−1^ and eV (bottom and top axes, respectively). The distributions for each photolysis wavelength are offset vertically for display purposes and the dotted lines in plots (A) and (C) show the square root of *P*(TKER) – normalised to the same maximum value – in order to accentuate the high TKER tails. The TKER_max_ values associated with the two-body fragmentation channels (4), (5) and (8) as well as the most exoergic three body fragmentations yielding CH_2_ and/or CH_3_ fragments (channels (12) and (6)) are also indicated by vertical arrows in panels (A) and (C). TKER_max_ values for other many-body fragmentation channels can be derived from Table 1 but, as noted in the text, given the assumed TOF to TKER conversion scheme it is likely that the products from such many-body fragmentations would appear at TKER values well below TKER_max_.

Fig. 4 presents the total kinetic energy release *P*(TKER) distributions (calculated on the basis that the partner to the observed fragment carries all of the remaining mass) and TKER-dependent best-fit recoil anisotropy (*β*) parameters^30^ obtained from analysing the ion images retrieved from the central time slice of the TOF-MS peaks corresponding to (A, B) CH_2_^+^ and (C, D) CH_3_^+^ ions recorded at FEL wavelengths *λ* = 125.6 nm (9.87 eV), 121.6 nm (10.19 eV), 118.2 nm (10.49 eV) and 112.0 nm (11.07 eV). Note that the signal intensities at TKER > 35 000 cm^−1^ are too low for recoil anisotropy parameters to be fitted satisfactorily. Fig. 4A and C also show the corresponding [*P*(TKER)]^1/2^ plots (dotted lines) to allow better visualisation of the high TKER data. The raw ion images are shown in Fig. S2 of the ESI.†

The distributions derived from the CH_2_^+^ ion images (Fig. 4A) assume that the co-fragments are CH_4_ (*i.e*. that the CH_2_ fragments derive from reaction (4) in Table 1). This assumption must be correct for the more translationally excited CH_2_ products, which display an anisotropic velocity distribution characterised by a positive recoil anisotropy parameter, *β* ∼ +0.5–0.7 (Fig. 4B), *i.e.* the CH_2_ fragments recoil preferentially along the axis parallel to the polarisation vector ***ε*** of the photolysis laser photons. But the *P*(TKER) distributions extend to TKER ∼0 – implying substantial internal excitation of some of the CH_2_ and/or CH_4_ fragments. Indeed, as Table 1 shows, the chosen photon energies exceed the thresholds for several three-body fragmentation processes that yield CH_2_ products. Some or all of channels (6), (13), (14) and (17) in Table 1 could contribute to the increased low-TKER product yield observed at the two shortest excitation wavelengths – a point to which we return later. Thus the precise form of the *P*(TKER) distribution at low TKER is ill-defined, since the momentum conservation arguments used to convert a measured CH_2_ fragment velocity (derived from the image radius) into a TKER value are likely not to apply in a three-body dissociation. But this does not negate the conclusions that (i) the relative yield of slow fragments in the CH_2_^+^ images increases with increasing photon energy and (ii) the slower fragments, which display minimal recoil anisotropy (*β* ∼ 0), likely arise *via* one or more of the three-body fragmentation processes.

The distributions derived from the CH_3_^+^ ion images (Fig. 4C) peak at TKER ∼0 and show a tail extending to higher TKER that becomes more anisotropic (to positive *β*) and relatively more intense as the photolysis wavelength is reduced. As can be deduced from Table 1, the maximum possible TKER of CH_3_ fragments formed *via* reaction (5) following excitation at *λ* = 121.6 nm (Fig. 4C) would be ∼6.38 eV (∼51 500 cm^−1^); the high-TKER tails of the *P*(TKER) distributions shown in Fig. 4C (derived assuming C–C bond fission) extend to values for which the direct C–C bond fission channel (5) is the only possible one photon induced CH_3_ fragment formation pathway. Most of the imaged CH_3_ fragments appear with much lower TKER, however. Table 1 shows several potential sources of slow CH_3_ radicals, including three-body dissociations (6), (12) and (14) and the production of an electronically excited CH_3_ partner (channel (16)), the relative likelihoods of which are discussed below.

### (c) H atom photofragment time-of-flight (TOF) spectra

**Fig. 5:**
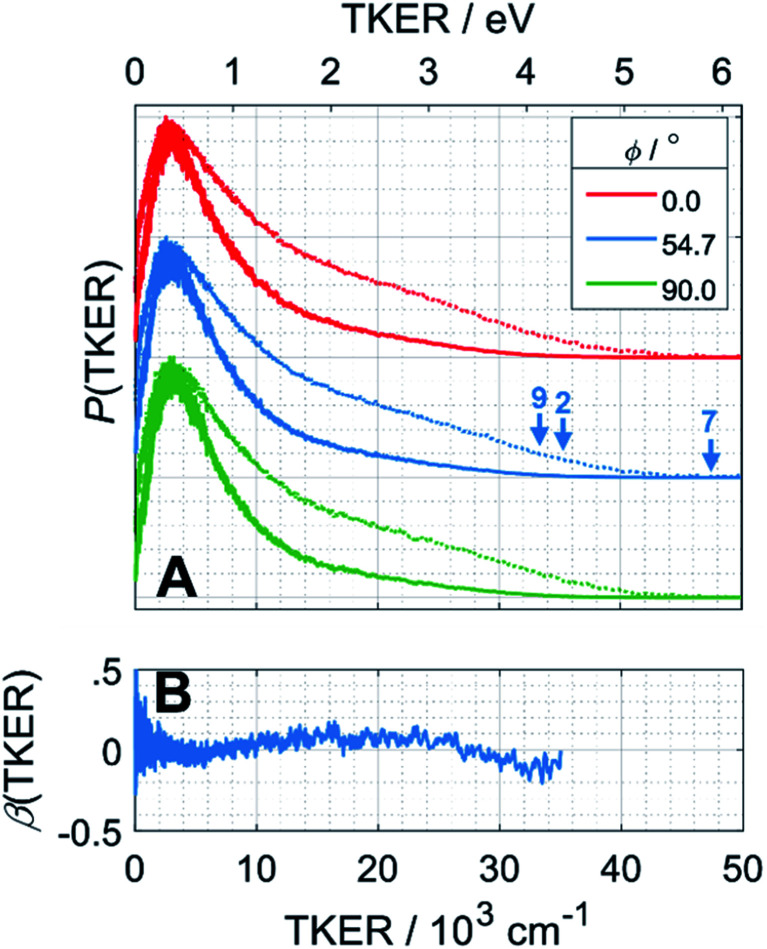
(A) *P*(TKER) distribution distributions derived from H atom TOF spectra recorded following photolysis of a jet-cooled C_2_H_6_ sample at *λ* = 121.6 nm with the ***ε*** vector aligned at *ϕ* = 0, 54.7 and 90° to the detection axis and (B) the *β*(TKER) distribution derived therefrom. As in Fig. 4, the individual data sets are offset vertically for display purposes and the high TKER part of the data are accentuated by also plotting [*P*(TKER)]^1/2^ distributions (dotted lines). The TKER_max_ values associated with primary C–H bond fission (channel (7)) and the two lowest energy three-body dissociation channels (2) and (9) from Table 1 are indicated by vertical arrows.

H atom TOF spectra were recorded following photolysis of a jet-cooled ethane sample in He at *λ* = 121.6 nm with ***ε*** aligned at, respectively, *ϕ* = 0, 54.7 and 90° to the detection axis and converted to the corresponding *P*(TKER) and *β*(TKER) distributions, shown in Fig. 5A and B, by assuming C_2_H_5_ as the co-fragment. The fastest products have TKER ∼35 000 cm^−1^ (∼4.3 eV). This TKER value is greater than that reported in the earlier imaging study at this wavelength^19^ but still well below the maximum allowed by energy conservation assuming single C–H bond fission in ethane (channel (7), for which TKER_max_ ∼ 6.5 eV). In contrast to the case of CH_4_, however, the H atom recoil velocity distribution is essentially isotropic.

### (d) Active photofragmentation channels

The present work identifies fragments formed by VUV photolysis of C_2_H_6_, assures that these arise *via* collision-free unimolecular dissociation and affords insights into the fragmentation dynamics. The translational spectroscopy data for the CH_2_, CH_3_ and H atom products hint at similarities in the fragmentation mechanisms following VUV photoexcitation of C_2_H_6_ and CH_4_ and it is useful to summarise current knowledge of the photofragmentation dynamics of CH_4_ to provide context for the discussion that follows.

Only the ground (S_0_) state and a repulsive triplet excited state of CH_4_ correlate with the lowest energy C–H bond fission limit (associated with H + CH_3_ products). The first excited singlet (S_1_) state of CH_4_ correlates adiabatically with 
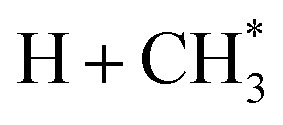
 products; the electronically excited 
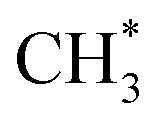
 fragments predissociate rapidly to H + ^1^CH_2_(ã) products.^31^ (Here and henceforth, we use superscript * and ^#^ symbols to indicate, respectively, electronically and rovibrationally excited products). Nonetheless, experiments find a substantial quantum yield of ground state C–H bond fission products following VUV photoexcitation of CH_4_, and the H atom products display anisotropic recoil velocity distributions – implying that the photoexcited molecules dissociate on a time scale that is much shorter than the rotational period of the parent molecule (which is estimated to be a few picoseconds).^30,32–34^ These findings highlight the importance of non-adiabatic couplings *via* conical intersection (CIs) between the S_1_ and S_0_ PESs.^35,36^ Theory shows that, to form H + CH_3_ products, one C–H bond in the photoexcited CH_4_ must start stretching and sweep through the plane defined by the other atoms to access the S_0_ PES and dissociate. Angular momentum conservation requires that the resulting CH_3_ products are highly rotationally excited; indeed, some of these CH_3_(X̃)^#^ fragments are formed with so much internal energy that they dissociate further – to H + CH_2_ and/or H_2_ + CH products. Rival distortions have also been identified, whereby photoexcited CH_4_ molecules dissociate by eliminating H_2_. Theory suggests that the partner CH_2_ fragments in this case are formed in the ã^1^A_1_ state (for dissociations occurring after non-adiabatic coupling to the parent S_0_ PES) and the b̃^1^B_1_ state (if dissociation occurs adiabatically on the excited state PES).^36^

Quantitative simulations of the early time nuclear motions following photoexcitation of C_2_H_6_ remain challenging but global reaction route mapping (GRRM)^37,38^ calculations (summarised in the ESI†) can offer important insights by predicting low-lying conical intersections (CIs) between the PESs for the S_0_ and S_1_ states. The present VUV photoexcitations will populate one or more S_*n*_ (*n* > 1) states of ethane, but we henceforth assume that molecules excited to these higher S_*n*_ states undergo efficient non-radiative coupling to the S_1_ state. As Fig. 2 showed, the S_1_ state of ethane correlates with 
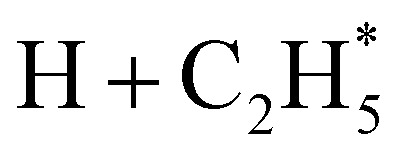
 and 
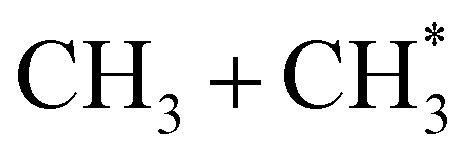
 products (channels (15) and (16) in Table 1). The 
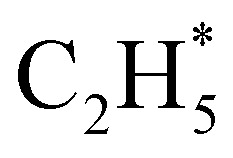
 and 
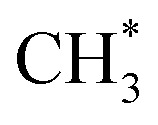
 species are both unstable and dissociate to give, respectively, H + C_2_H_4_ (ref. 39–41) and H + ^1^CH_2_(ã) (ref. 31) products. As in CH_4_, C_2_H_6_(S_1_) molecules can also decompose after non-adiabatic coupling to the S_0_ PES. The nuclear distortions required to access the predicted CIs between the S_1_ and S_0_ PESs (shown in Fig. 2) correlate well with ^1^CH_2_ elimination once an H atom has inserted between the two C atoms, with C–C or C–H bond fissions and with loss of H_2_. The present study is sensitive to the first three fragmentation processes, which are considered in turn. Given the photon energies involved and the multi-dimensional nature of many of these distortions, we can anticipate that (as in the case of CH_4_ (ref. 30, 33 and 34)) many of the polyatomic products will be formed with sufficient internal energy that they will fragment further.

#### CH_2_ radical formation

The imaging studies reveal CH_2_ fragments, with non-zero *β* values, implying that these are again formed on a timescale shorter than the parent rotational period. The *P*(TKER) distributions extend to values where the partner fragment can only be CH_4_, but not to sufficiently high TKER values to allow unambiguous determination of the electronic state of the CH_2_. Spin-conservation arguments suggest that CH_2_ radicals formed in tandem with CH_4_ will be in their ã^1^A_1_ state (for dissociations that occur following non-adiabatic coupling at a CI with the S_0_ PES) and/or b̃^1^B_1_ state (for dissociation on the S_1_ PES). But the distributions also extend to TKER ∼0, showing that one or other or both fragments are formed with a broad spread of internal energies. The photoexcitation energies are sufficient to induce three-body fragmentations and, simply on energetic grounds, any of channels (6), (13), (14) and (17) in Table 1 could contribute to signal in the CH_2_^+^ ion images. Of these, unimolecular decay of any sufficiently internally excited CH^#^_4_ partner would be expected to contribute to the yield of (slow) H and CH_3_ products, *i.e.* the net reaction (14) in Table 1.

Fig. 4A shows an additional feature at low TKER in the distributions derived from the CH_2_^+^ images measured at the two shorter wavelengths. This might signify the opening of a new (three-body) route to ^1^CH_2_ products, but this feature more likely indicates the presence of ^3^CH_2_ photoproducts: The ground states of the ^3^CH_2_ radical and the CH_2_^+^ cation have very similar geometries. Photoionisation of ^3^CH_2_ thus tends to be vibrationally adiabatic (*i.e*. to favour Δ*v* = 0 transitions)^42–44^ and, from Table S1,† should only be expected at *E*_phot_ >10.39 eV (*i.e*. at *λ* < 119.3 nm). Note that the feature at low TKER in the *P*(TKER) distributions shown in Fig. 4A appears to ‘turn on’ and become more prominent as the photon energy is tuned above this threshold. Several possible routes to forming ^3^CH_2_ products can be envisaged, including the unimolecular decay of highly internally excited CH^#^_3_ fragments (from initial C–C bond fission) or of C_2_H^#^_5_ fragments (following primary C–H bond fission) after non-adiabatic coupling to the S_0_ state – as discussed below. Both would contribute to net process (6) in Table 1, though not necessarily exhibit similar energy disposals.

#### C–C bond fission

The tails of the *P*(TKER) distributions derived from the CH_3_^+^ images extend to TKER values that can only be accommodated by assuming C–C bond fission and formation of two CH_3_ radicals (*i.e*. reaction (5) in Table 1). Most of the measured CH_3_ fragment velocities imply TKER values far below the maximum allowed by energy conservation, however. Focussing first on the high TKER region in Fig. 4C, the CH_3_ fragment yield is clearly rising with decreasing TKER, indicating a preference that one (or both) CH_3_ fragments from reaction (5) are formed internally excited. Such energy disposal would likely be a consequence of the nuclear motions that enable non-adiabatic coupling to the S_0_ PES. Again, the non-zero *β* parameter revealed by the CH_3_ images imply that these nuclear motions and the ensuing C–C bond fission on the S_0_ PES also occur on a timescale shorter than the parent rotational period.

In principle, the entire *P*(TKER) distribution derived from the CH_3_ image could be attributed to channel (5) if the fragmentation dynamics were heavily biased towards forming very highly internally excited CH^#^_3_ products. The unimolecular decay of these CH^#^_3_ fragments would be a source of the ^3^CH_2_ products inferred above (reaction (6)), and these ^3^CH_2_ products would be expected to display a similar translational energy distribution to that of the CH^#^_3_ products (since the light H atom partner would take the bulk of any excess energy released in the secondary fragmentation). Such expectations are consistent with the experimental data and, as noted above, the non-observation of a peak attributable to ^3^CH_2_ products at longer wavelengths (*e.g.* at *λ* = 121.6 nm) need not imply that CH^#^_3_ fragments are not formed but simply that the ^3^CH_2_ products from their decay are not amenable to photoionisation at the longer wavelengths.

The dominance of translationally ‘cold’ (*i.e*. internally ‘hot’) CH_3_ products in the *P*(TKER) distributions is striking, however. Table 1 suggests several other potential sources of slow CH_3_ products. Adiabatic dissociation on the S_1_ PES to 
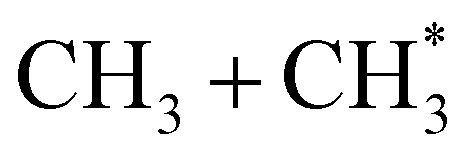
 products is an interesting contender. This process is exoergic at all wavelengths studied, though the adiabatic S_1_ PES will likely exhibit a barrier at short *R*_C–C_ bond extensions as the Rydberg function acquires increasing σ* antibonding valence character.^6^ The 
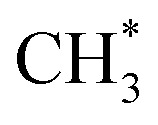
 radicals would be unstable with respect to H + ^1^CH_2_(ã) products.^31^ Again, the H atoms would carry most of any kinetic energy release, so the translational energy distributions of any ^1^CH_2_(ã) fragments formed in this way should broadly mirror that of their 
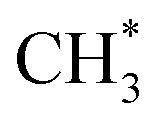
 precursor. ^1^CH_2_(ã) fragments are amenable to photoionisation at all wavelengths investigated in the present work, but the TKER distributions derived from the CH_2_^+^ images measured at the longer excitation wavelengths show no ‘spike’ at low TKER – suggesting that any contribution to the ^1^CH_2_(ã) yield from adiabatic dissociation to 
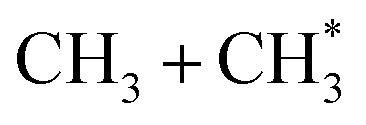
 products on the S_1_ PES must be small compared to that from reaction (4).

### C–H bond fission

The *P*(TKER) distribution derived from the H atom TOF measurements (Fig. 5A) extends to TKER values that can only be attributed to prompt C–H bond fission following VUV photoexcitation of C_2_H_6_, *i.e*. to reaction (7) in Table 1. The C_2_H_5_ co-fragments are formed with a very broad spread of internal energies. Analogy with CH_4_ suggests that this energy disposal is a consequence of the nuclear motions that promote C–H bond fission by non-adiabatic coupling to the S_0_ PES.^6,35^ Most of the ‘C_2_H_5_’ products assumed in deriving the *P*(TKER) distribution have sufficient internal energy to dissociate further – by loss of another H atom (net reaction (2)), or H_2_ (net process (9)), or both (net channel (10)), or to two CH_*x*_ species (*e.g*. *via* net channels (6) or (11) in Table 1).^42^ However, the smoothly varying *P*(TKER) distributions shown in Fig. 5A suggest that such overall three- (or more-) body dissociations occur sequentially, *i.e. via* a prompt C–H bond fission and subsequent unimolecular decay of the resulting C_2_H^#^_5_ radicals. The low-TKER peak in Fig. 5A could also indicate an adiabatic contribution to the overall dissociation, yielding 
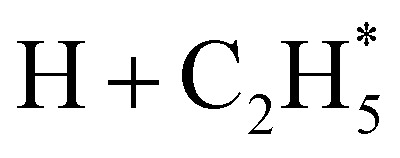
 as primary products – as suggested in the earlier imaging study at *λ* = 121.6 nm.^19^ Any 
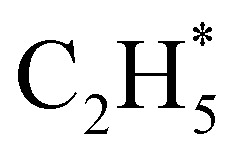
 fragments would dissociate, yielding H + C_2_H_4_ products with a spread of translational energies^39,45^ (*i.e*. net reaction (2)). Many of the C_2_H_4_ products formed by decay of C_2_H^#^_5_ or 
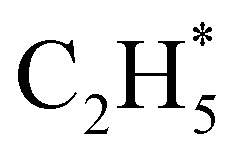
 species may well be formed with sufficient internal energy to decay yet further, to H + C_2_H_3_ (vinyl) radical products, or by eliminating H_2_ to yield C_2_H_2_. The former products are observed in the present study, *via* the C_2_H_3_^+^ peak in the TOF-MS in Fig. 3 and corresponding small ion image shown in Fig. S3 of the ESI,† but the current work is blind to C_2_H_2_ products – which were identified by end-product analysis in the early VUV photolysis studies of C_2_H_6_ under collisional conditions.^16–18^ For completeness, we note that C_2_H_2_ products could also arise *via* sequential H_2_ eliminations from, first, C_2_H^#^_6_ (formed by non-adiabatic coupling to the S_0_ state) and then from the resulting C_2_H^#^_4_ intermediates (*i.e.* net reaction (3) in Table 1). C_2_H_2_ formation by loss of four H atoms from C_2_H_6_ is energetically forbidden at the VUV wavelengths of current interest.

The present study does not return quantum yields and, as noted above, is silent regarding some molecular elimination channels. But it certainly identifies several active fragmentation channels and provides new insights into the likely fragmentation dynamics. The present analysis finds no compelling evidence for adiabatic dissociation on an excited state PES – implying efficient non-adiabatic coupling between excited states of C_2_H_6_ and to the S_0_ PES. Many of the present interpretations align with the results of recent quasi-classical trajectory surface hopping calculations for the next larger alkane, propane (C_3_H_8_), following excitation at *λ* = 157 nm, wherein it was concluded that most dissociations occur after internal conversion to the S_0_ PES, that the energy disposal in the resulting fragments is governed by dynamical rather than statistical factors, and that the three-body fragmentation processes occur sequentially.^46^

### (e) Implications for modelling the atmospheres of the gas giants

This work provides detailed new insights into the VUV photochemistry of ethane. The results discussed in detail in the above subsections are summarised below in terms of their implications for modelling the atmospheres of the gas giants. These new results must influence future models:

(i) The HRA-PTS studies reveal kinetic energy distributions extending to values that, on energetic grounds, can only be attributed to prompt C–H bond fission, confirming primary C–H bond fission yielding H + C_2_H_5_ products (reaction (7) in Table 1). This reaction does not feature in current models used to describe the chemical processing in the stratospheres of the gas giants. Most of the C_2_H_5_ species are formed with enough internal energy to decay further, almost certainly yielding some H + C_2_H_4_ products. The present study thus supports inclusion of reaction (2) in the modelling and implies that the two H atoms in that case are lost sequentially.

(ii) The kinetic energy distributions derived from the CH_3_^+^ ion images extend to TKER values that can only be attributed to C–C bond fission yielding two CH_3_ radicals, confirming that the C–C bond fission channel (reaction (5)) is active and supporting its inclusion in the modelling. The finding that the *P*(TKER) distributions peak at TKER ∼0 implies that one of the CH_3_ fragments is generally formed with sufficient internal energy to decay further. If C–C bond fission completes after non-adiabatic coupling to the S_0_ PES, the resulting CH^#^_3_ fragments most likely decay to H + ^3^CH_2_(X̃) products (*i.e*. net reaction (6)). This reaction is not included in the current model and, according to the present analysis, will have significantly higher quantum yield than reaction (5).

(iii) The imaging studies confirm formation of CH_2_ fragments, with a smooth *P*(TKER) distribution that extends to TKER values such that the partner fragment can only be CH_4_. Spin-conservation arguments and the deduced efficiency of non-adiabatic coupling to the S_0_ PES suggest that these faster CH_2_ fragments are formed in the ã^1^A_1_ state. The inclusion of reaction (4) in the photochemical modelling is vindicated.

(iv) The primary fragmentations and resulting product energy disposals following VUV photoexcitation of ethane are shown to be governed by dynamical rather than statistical factors; three-body dissociations are commonplace and occur sequentially. Clearly, quantitative branching ratios for the various active channels are still needed, but the present work offers several clear pointers. Reaction (7) and, particularly, the three-body fragmentation (6) are active and require incorporation in future modelling. The yield of (currently neglected) reaction (6) is deduced to be larger than that of reaction (5). The processes revealed in this study all involve relatively ‘prompt’ C–H or C–C bond fission, after non-adiabatic coupling to the S_0_ PES. As Fig. 2 shows, the respective bond energies are lower than the energy barriers to C_2_H_4_ formation by H_2_ elimination on the S_0_ PES. Analogy with CH_4_ suggests that any H_2_ and C_2_H_4_ products formed *via* process (1) will be both translationally and vibrationally excited. The likelihood that the C_2_H^#^_4_ species would have sufficient internal energy to surmount the barrier to eliminating a further H_2_ (to yield C_2_H_2_ or H_2_CC) is unclear. We further note that the substantial (∼60%) branching into C_2_H_*x*_ species following VUV photoexcitation of C_2_H_6_ assumed in the current planetary atmospheric photochemistry models derives from indirect measurements made more than half a century ago, and is predicated on an assumption that the decomposition of the internally excited C_2_H^#^_4_ species formed *via* reaction (1) would mimic that deduced following VUV photoexcitation of strategically deuterated ethene (CH_2_CD_2_) molecules.^16,17^ Such an assumption must be questionable, given the differences in available energy and the recognised importance of dynamics (*i.e*. the topographies of, and non-adiabatic couplings between, the PESs sampled in the two cases) in determining the product branching and energy disposal. It seems likely that the current models overestimate the relative yield of C_2_H_*x*_ (particularly C_2_H_2_) photoproducts.

## Conclusions

Translational spectroscopy methods employing two cutting-edge technologies – the Dalian Coherent Light Source (DCLS) Free Electron Laser (FEL) and a fast-framing PImMS2 camera – have revealed many new insights into the rich (and hitherto largely impenetrable) VUV photochemistry of ethane. The present findings should serve to stimulate *ab initio* molecular dynamics simulations of this prototypical alkane following photoexcitation at VUV wavelengths and substantial refinements of the models currently used to describe the atmospheric photochemistry of the gas giants. This study (i) concludes that, as in CH_4_, the VUV photochemistry of ethane is driven by efficient non-adiabatic coupling to, and subsequent direct (and sequential) dissociations on the S_0_ PES, (ii) highlights the need to revise current photochemical models of the stratospheric photochemistry of the gas giants – by incorporating the hitherto neglected C–H bond fission channel (7) and the three-body decomposition (6) to CH_3_ + ^3^CH_2_ + H products and down-grading the relative yield of primary C_2_H_*x*_ photoproducts – and (iii) emphasises the pressing need for quantitative product branching fractions. Stratospheric C_2_H_6_ production in the gas giants is driven by VUV photodissociation of CH_4_, but the present analysis implies that the subsequent photochemical coupling between C_2_H_6_ and C_2_H_2_ is likely to be weaker than currently assumed.

## Data and materials availability

The raw ion event lists, H-atom TOF spectra and calculation log files are available from the authors upon reasonable request.

## Conflicts of interest

There are no conflicts to declare.

## Supplementary Material

SC-011-D0SC01746A-s001
