# Supplementary material for: Ultraviolet photochemistry of ethane: implications for the atmospheric chemistry of the gas giants
Source: Chem Sci. 2020 Apr 29;11(19):5089–97. doi: 10.1039/d0sc01746a (PMC8159213; doi:10.1039/d0sc01746a)
Supplement: SC-011-D0SC01746A-s001 [file SC-011-D0SC01746A-s001.pdf]

Electronic Supplementary Material (ESI) for Chemical Science.
This journal is © The Royal Society of Chemistry 2020

**Supporting Information (SI) to accompany**

# Ultraviolet photochemistry of ethane: implications for the atmospheric chemistry of the gas giants.

Yao Chang,[a] Jiayue Yang,[a] Zhichao Chen,[a] Zhiguo Zhang,[a,b] Yong Yu,[a] Qingming Li,[a] Zhigang He,[a] Weiqing Zhang,[a] Guorong Wu,[a] Rebecca A. Ingle,[c] Matthew Bain,[d] Michael N.R. Ashfold,[d]* Kaijun Yuan,[a]* Xueming Yang,[a,e]* Christopher S. Hansen.[f]*

a. State Key Laboratory of Molecular Reaction Dynamics, Dalian Institute of Chemical Physics, Chinese Academy of Sciences, 457 Zhongshan Road, Dalian, 116023, China.
b. Key Laboratory of Functional Materials and Devices for Informatics of Anhui Higher Education Institutions and School of Physics and Electronic Engineering, Fuyang Normal University, Fuyang, Anhui 236041, China
c. Department of Chemistry, University College London, London, WC1H 0AJ, UK
d. School of Chemistry, University of Bristol, Bristol, BS8 1TS, U.K.
e. Department of Chemistry, Southern University of Science and Technology, Shenzhen 518055, China
f. School of Chemistry, University of New South Wales, Sydney, NSW 2052, Australia

**Authors for correspondence:**

Christopher S. Hansen, Kaijun Yuan, Xueming Yang, Michael N.R. Ashfold

**Email:**

christopher.hansen@unsw.edu.au

kjyuan@dicp.ac.cn

xmyang@dicp.ac.cn

mike.ashfold@bristol.ac.uk

## Experimental methods

The imaging experiments all employed a recently constructed apparatus for molecular photochemistry centered on the vacuum ultraviolet free electron laser (VUV-FEL) beam line at the Dalian Coherent Light Source (DCLS).[1] The VUV-FEL facility runs in the high gain harmonic generation (HGHG) mode,[2] wherein a seed laser is injected to interact with an electron beam in the modulator. The seed pulse, with a wavelength in the range $240 \leq \lambda_{seed} \leq 360$ nm, is generated from a Ti:sapphire laser, while the electron beam is produced from a photocathode RF gun and accelerated to a beam energy of ~300 MeV by 7 S-band accelerator structures, with a bunch charge of 500 pC. The micro-bunched beam is sent through the radiator, which is tuned to the *n*th harmonic ($n$ = 2-4) of $\lambda_{seed}$, yielding coherent FEL radiation with wavelength $\lambda_{seed}/n$. After optimisation, the linear accelerator can yield pulse energies >200 μJ, with duration ~1.5 ps, at a repetition rate of 10 Hz at any user selected wavelength in the range $50 \leq \lambda \leq 150$ nm with typical spectral bandwidths in the range 30-70 $cm^{-1}$.

The molecular beam for the ion imaging experiments was generated by expanding a mixture of $C_2H_6$ and He at a total pressure of 600-900 Torr through a 0.5 mm diameter pulsed nozzle (General Valve), which was skimmed before entering the differentially pumped interaction region between the repeller and extractor electrodes of a 23 plate ion optics assembly. The molecular beam propagates along the $z$-axis (*i.e.* vertically upwards in the laboratory frame), along the centre axis of the ion optics assembly and strikes the centre of a 75 mm diameter microchannel plate (MCP) detector. The FEL radiation propagates at right angles to the molecular beam axis (along the $x$-axis) and is linearly polarised with its **ε** vector aligned along the $y$-axis. The cations formed in the interaction region are accelerated along the $z$-axis and enter a 740 mm field-free drift region wherein they separate in time according to their mass to charge ($m/z$) ratio before striking the MCP detector coupled to a P47 phosphor screen (Photek VID275). The detector gain was time-gated to allow selection of just a portion of the ion TOF spectrum and the screen imaged by a PImMS2 sensor[3, 4] through a Nikon NIKKOR 55 mm macro-lens ($f$/2.8). The time resolution of the PImMS2 sensor was set to 25 ns which, for the chosen ion optics voltages, provided 6-8 time slices through each $m/z$ peak of interest. The data were processed by centroiding the ($x$, $y$, $t$) event lists associated with each laser shot in space and time to reduce event clusters to single ion events as described previously.[5-7] TOF spectra could be created at this point by summing all events arriving in each time bin and converted to an $m/z$ spectrum by comparison with the TOFs of a known set of masses. Ion images

representing the central slice of each $m/z$ ion ensemble were constructed by plotting the $(x, y)$ coordinates of events within the single time bin corresponding to the peak TOF signal for the appropriate $m/z$. This has been shown to give equivalent results to 'crushing' the full TOF peak and mathematically recovering the central slice.[5] The recoil anisotropy parameter ($\beta$) of any feature of interest was determined by fitting to Eq. (1),

$$I(\theta) \propto 1 + \beta P_2(\cos\theta). \qquad (1)$$

where $P_2(\cos\theta)$ is the second order Legendre polynomial and $\theta$ is the angle between the polarisation vector of the photolysis laser radiation, $\boldsymbol{\varepsilon}$, and the recoil velocity. The radius to velocity calibration was achieved measuring the known velocity distribution of $O^+$ cations formed following the photoexcitation of $O_2$ at $\lambda = 225.67$ nm.[8]

The H-atom TOF data reported here were obtained using more traditional, table-top nanosecond pulsed laser systems. The Rydberg tagging method[9, 10] relies on 1(VUV)+1′(UV) excitation of H atom fragments. The first step involves H($n$=2 ← $n$=1) excitation by absorption of a $\lambda = 121.57$ nm photon. The H($n$=2) atom is then excited further, with a $\lambda$ ~365 nm photon, to a high-$n$ ($n$ = 30-80) Rydberg state. The $\lambda = 121.57$ nm photons were generated by four wave difference frequency mixing using two 212.556 nm photons and one ~845 nm photon in a cell containing a Kr/Ar gas mixture (1:3 mixing ratio). The 212.556 nm laser light for the latter source was produced by frequency doubling the output of a tunable dye laser operating at $\lambda$ ~425 nm, pumped by the third harmonic output of a pulsed Nd:YAG laser. The 845 nm laser light was the direct output of another dye laser pumped by part of the second harmonic output from the same Nd:YAG laser. Both laser beams were linearly polarised, and the latter passed through a half-waveplate *en route* to the four wave mixing cell to allow rotation of its polarisation vector, $\boldsymbol{\varepsilon}$, (and thus the polarisation of the generated Lyman-$\alpha$ radiation). As shown in the paper, VUV photolysis of ethane yields alkyl (methyl, ethyl) radical products. These are readily photodissociated (yielding further H atoms) when excited at 212.556 nm.[11] Any contribution from such unintended secondary photolysis was minimised by arranging that the 212.556 nm, 845 nm and the generated 121.57 nm beams passed through an off-axis biconvex LiF lens at the end of the mixing cell. The dispersion of the LiF lens then ensured the required spatial separation of the 121.57 and 212.556 nm radiation in the interaction region. The 365 nm laser radiation, required whatever the source of the Lyman-$\alpha$ radiation, was generated by doubling the output of a tunable dye laser operating at $\lambda$ ~730 nm, pumped by another fraction of the second harmonic output of the Nd:YAG laser.

The Rydberg tagged H atom photofragments are detected by a smaller (40 mm diameter) MCP detector positioned close behind a grounded metal mesh at a distance $d \approx 280$ mm from the interaction region. After passing through the mesh, the Rydberg atoms are immediately field-ionised by the electric field (~2000 V $cm^{-1}$) between the grid and the front plate of the detector. Any charged species formed in the Rydberg tagging experiments were extracted from the TOF axis by applying a small (~20 V $cm^{-1}$) electric field perpendicular to the TOF axis. The signal from the MCP was passed by a fast pre-amplifier and counted using a multichannel scaler. TOF spectra along axes parallel (*i.e.* along $y$), perpendicular (along $z$) and at the magic angle (54.7°) to **ε** were measured by rotating the MCP detector and subsequently converted to $P$(TKER) spectra as described in the main text.

**Computation methods**

Minimum energy conical intersections (MECIs) – an illustrative sub-set of which are presented in Fig. 1 – were located using the seam model function (SMF)/single-component artificial force induced reaction (SC-AFIR) method[12, 13] as implemented in a developmental version of the global reaction route mapping (GRRM) code.[14] Calculations were performed using spin-flip time-dependent density functional theory (SF-TDDFT) with the BHHLYP functional[15] and a 6-31G(d) basis set as implemented in GAMESS.[16] The ground state equilibrium structure calculated using density functional theory with the same functional and basis set was used as a starting point for the search.

| Species | Ionisation potential / eV |
|---|---|
| $C_2H_6$ | 11.49 |
| $C_2H_5$ | 8.13 |
| $C_2H_4$ | 10.51 |
| $C_2H_3$ | 8.48 |
| $C_2H_2$ | 11.40 |
| | |
| $CH_4$ | 12.62 |
| $CH_3$ | 9.84 |
| $^1CH_2(a)$ | 10.00 |
| $^3CH_2(X)$ | 10.39 |
| CH | 10.64 |

**Table S1.** Adiabatic ionisation energies of selected species of interest, calculated using $\Delta_f H$ (0 K) values from ref. [17].

| Species | Fragments | Dissociative ionisation threshold / eV |
|---|---|---|
| $C_2H_6$ | $CH_3 + CH_3^+$ | 13.65 |
| | $CH_4 + CH_2^+$ | 14.46 |
| | $^3CH_2 + CH_4^+$ | 16.69 |
| | | |
| $C_2H_5$ | $^3CH_2 + CH_3^+$ | 14.09 |
| | $CH_3 + CH_2^+$ | 14.64 |
| | | |
| $CH_4$ | $H + CH_3^+$ | 14.32 |
| | $H_2 + CH_2^+$ | 15.13 |
| | | |
| $CH_3$ | $H + CH_2^+$ | 15.04 |
| | | |
| $^3CH_2$ | $H + CH^+$ | 15.00 |
| $^1CH_2$ | $H + CH^+$ | 14.61 |

**Table S2.** Adiabatic dissociative ionisation energies for processes yielding (or depleting) selected $C_1H_x^+$ species of interest, calculated using $\Delta_f H$ (0 K) values from Ref. [17].

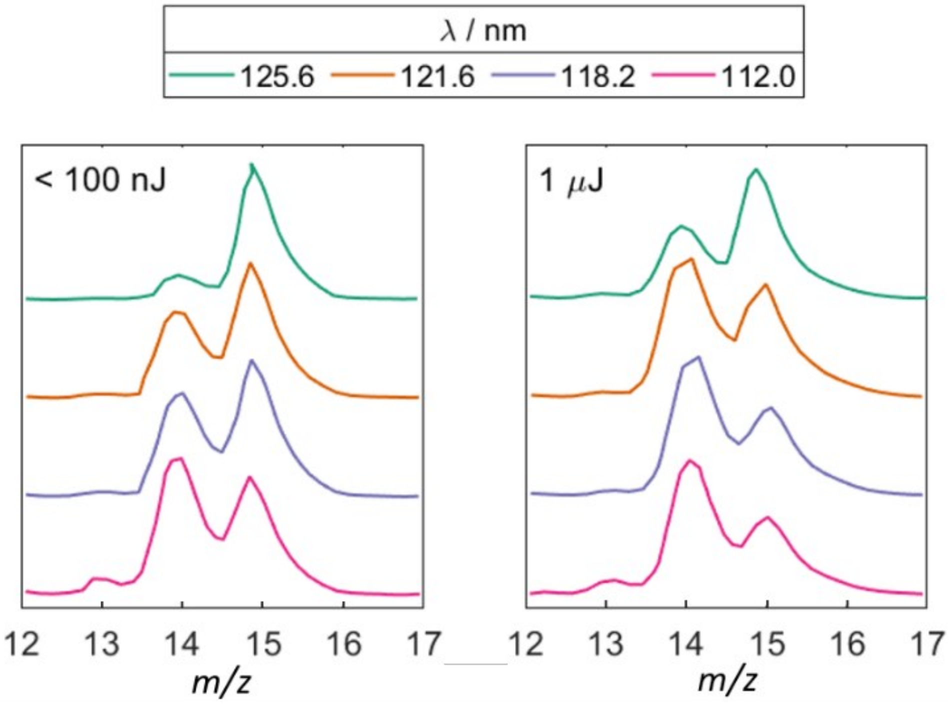


**Figure S1**. Mass spectra (derived from measured TOF spectra of ions formed by universal ionisation) showing the $CH_2$ (*m/z* 14) and $CH_3$ (*m/z* 15) products from photolysis of $C_2H_6$ at $\lambda$ = 125.6, 121.6, 118.2 and 112.0 nm using two different FEL pulse energies (< 100 nJ and 1 μJ), offset vertically for ease of display. The relative intensity of the $CH_2$ peak increases with decreasing excitation wavelength (*i.e.* with increasing $E_{phot}$) and with increasing pulse energy (which we ascribe to an increased relative ionisation probability). All data reported in the main paper were obtained at the lowest FEL pulse energies compatible with stable signal levels.

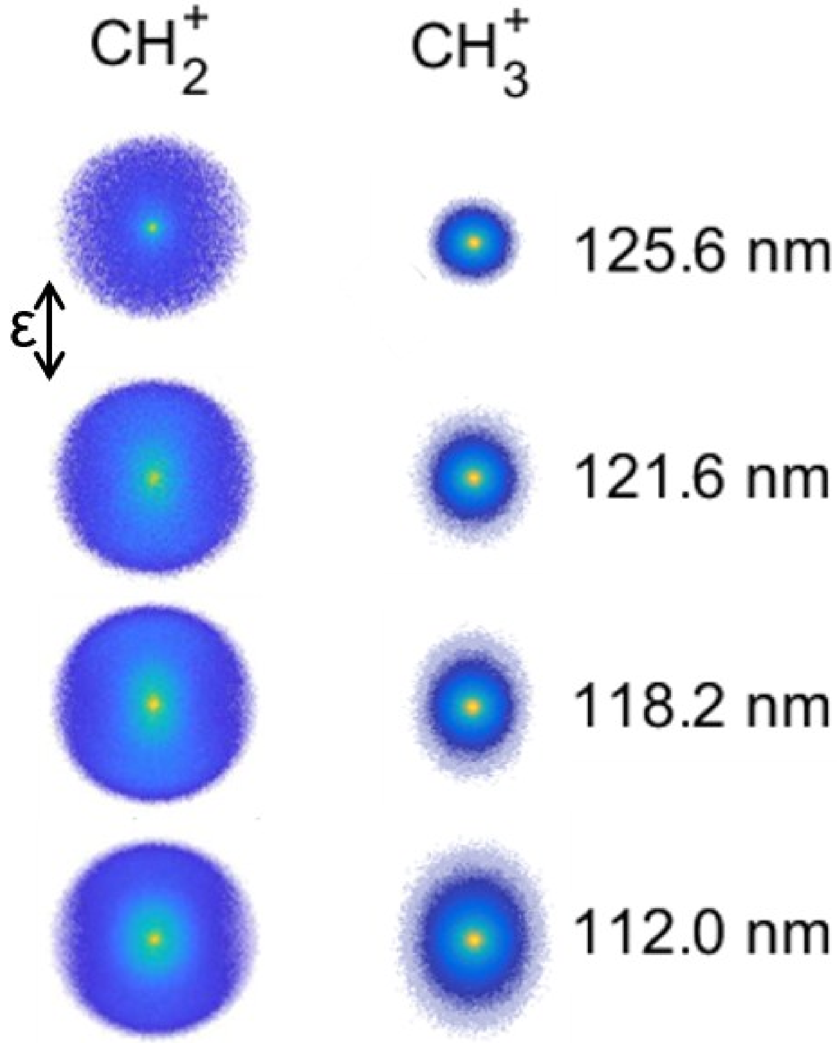


**Figure S2.** Raw $CH_2^+$ and $CH_3^+$ ion images (central time slices) analysed to prepare the *P*(TKER) and *β*(TKER) distributions in Figure 4 of the main text. The FEL excitation wavelength is shown to the right of each $CH_2^+$/$CH_3^+$ ion image pair and the polarisation (**ε**) vector of the FEL excitation light was vertical in the plane of the figure as indicated by the double headed arrow at the top left.

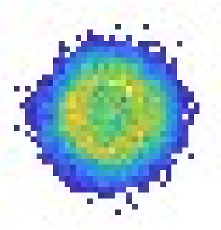

**Figure S3.** Enlarged (arbitrarily) raw $C_2H_3^+$ ion image (central time slice) acquired following $\lambda$ = 121.6 nm (FEL) photolysis of a jet-cooled $C_2H_6$ sample. Note that, unlike the ion images in Fig. S2, this ion image results from a dissociation channel wherein an undetected, lighter-mass fragment (or fragments) receives most of the released translational energy (by conservation of momentum). All ion images in this study were acquired at a fixed set of electrode voltages, chosen such that the $CH_2^+$ ion image would fill most of the detector, and thus the $C_2H_3^+$ ion image (recoiling from $H_2$ or H) is small. The polarisation (**ε**) vector of the FEL excitation light was vertical in the plane of the figure as in Fig. S2.

## References


1. Y. Chang, S. Yu, Q. Li, Y. Yu, H. Wang, S. Su, Z. Chen, L. Che, X. Wang, W. Zhang, D. Dai, G. Wu, K. Yuan and X. Yang, *Review of Scientific Instruments*, 2018, **89**, 063113.
2. L. H. Yu, M. Babzien, I. Ben-Zvi, L. F. DiMauro, A. Doyuran, W. Graves, E. Johnson, S. Krinsky, R. Malone, I. Pogorelsky, J. Skaritka, G. Rakowsky, L. Solomon, X. J. Wang, M. Woodle, V. Yakimenko, S. G. Biedron, J. N. Galayda, E. Gluskin, J. Jagger, V. Sajaev and I. Vasserman, *Science*, 2000, **289**, 932-935.
3. J. J. John, M. Brouard, A. Clark, J. Crooks, E. Halford, L. Hill, J. W. L. Lee, A. Nomerotski, R. Pisarczyk, I. Sedgwick, C. S. Slater, R. Turchetta, C. Vallance, E. Wilman, B. Winter and W. H. Yuen, *Journal of Instrumentation*, 2012, **7**, C08001.
4. A. T. Clark, J. P. Crooks, I. Sedgwick, R. Turchetta, J. W. L. Lee, J. J. John, E. S. Wilman, L. Hill, E. Halford, C. S. Slater, B. Winter, W. H. Yuen, S. H. Gardiner, M. L. Lipciuc, M. Brouard, A. Nomerotski and C. Vallance, *Journal of Physical Chemistry A*, 2012, **116**, 10897-10903.
5. K. Amini, S. Blake, M. Brouard, M. B. Burt, E. Halford, A. Lauer, C. S. Slater, J. W. L. Lee and C. Vallance, *Review of Scientific Instrumentation*, 2015, **86**, 103113.
6. R. A. Ingle, C. S. Hansen, E. Elsdon, M. Bain, J. W. L. Lee, M. Brouard, C. Vallance, R. Turchetta and M. N. R. Ashfold, *Journal of Chemical Physics*, 2017, **147**, 013914.
7. M. Bain, C. S. Hansen and M. N. R. Ashfold, *Journal of Chemical Physics*, 2018, **149**, 081103.
8. B. Buijsse, W. J. v. d. Zande, A. T. J. B. Eppink, D. H. Parker, B. R. Lewis and S. T. Gibson, *Journal of Chemical Physics*, 1998, **108**, 7229-7243.
9. L. Schnieder, W. Meier, K. H. Welge and M. N. R. A. C. M. Western, *Journal of Chemical Physics*, 1990, **92**, 7027-7037.
10. M. N. R. Ashfold, K. J. Yuan and X. M. Yang, *Journal of Chemical Physics*, 2018, **149**, 080901.
11. M. N. R. Ashfold, R. A. Ingle, T. N. V. Karsili and J. S. Zhang, *Physical Chemistry Chemical Physics*, 2019, **21**, 13880-13901.
12. S. Maeda, T. Taketsugu, K. Ohno and K. Morokuma, *Journal of the American Chemical Society*, 2015, **137**, 3433-3445.
13. S. Maeda, T. Taketsugu and K. Morokuma, *Journal of Computational Chemistry*, 2014, **35**, 166-173.
14. S. Maeda, Y. Osada, Y. Harabuchi, T. Taketsugu, K. Morokuma and K. Ohno, *GRRM, a developmental version*, Hokkaido University, Sapporo, 2015.
15. A. D. Becke, *Journal of Chemical Physics*, 1993, **98**, 1372-1377.
16. M. W. Schmidt, K. K. Baldridge, J. A. Boatz, S. T. Elbert, M. S. Gordon, J. H. Jensen, S. Koseki, N. Matsunaga, K. A. Nguyen, S. Su, T. L. Windus, M. Dupuis and J. A. Montgomery, *Journal of Computational Chemistry*, 1993, **14**, 1347-1363.
17. B. Ruscic and D. H. Bross, Active Thermochemical Tables (ATcT) values based on ver. 1.122g of the Thermochemical Network (2019); available at ATcT.anl.gov).
